# Cardiovascular Disease Risk Models and Longitudinal Changes in Cognition: A Systematic Review

**DOI:** 10.1371/journal.pone.0114431

**Published:** 2014-12-05

**Authors:** Stephanie L. Harrison, Jie Ding, Eugene Y. H. Tang, Mario Siervo, Louise Robinson, Carol Jagger, Blossom C. M. Stephan

**Affiliations:** 1 Institute of Health and Society, Newcastle University, Newcastle upon Tyne, Tyne and Wear, United Kingdom; 2 Laboratory of Epidemiology and Population Sciences, National Institute on Aging, NIH, Bethesda, Maryland, United States of America; 3 Institute for Ageing and Health, Newcastle University, Newcastle upon Tyne, Tyne and Wear, United Kingdom; Univ. Kentucky, United States of America

## Abstract

**Background:**

Cardiovascular disease and its risk factors have consistently been associated with poor cognitive function and incident dementia. Whether cardiovascular disease prediction models, developed to predict an individual's risk of future cardiovascular disease or stroke, are also informative for predicting risk of cognitive decline and dementia is not known.

**Objective:**

The objective of this systematic review was to compare cohort studies examining the association between cardiovascular disease risk models and longitudinal changes in cognitive function or risk of incident cognitive impairment or dementia.

**Materials and Methods:**

Medline, PsychINFO, and Embase were searched from inception to March 28, 2014. From 3,413 records initially screened, 21 were included.

**Results:**

The association between numerous different cardiovascular disease risk models and cognitive outcomes has been tested, including Framingham and non-Framingham risk models. Five studies examined dementia as an outcome; fourteen studies examined cognitive decline or incident cognitive impairment as an outcome; and two studies examined both dementia and cognitive changes as outcomes. In all studies, higher cardiovascular disease risk scores were associated with cognitive changes or risk of dementia. Only four studies reported model prognostic performance indices, such as Area Under the Curve (AUC), for predicting incident dementia or cognitive impairment and these studies all examined non-Framingham Risk models (AUC range: 0.74 to 0.78).

**Conclusions:**

Cardiovascular risk prediction models are associated with cognitive changes over time and risk of dementia. Such models are easily obtainable in clinical and research settings and may be useful for identifying individuals at high risk of future cognitive decline and dementia.

## Introduction

Modification of health and lifestyle factors could improve vascular heath and possibly reduce the risk of cognitive decline and dementia [1]. Indeed, modification of health and lifestyle factors such as, diabetes, hypertension, obesity, physical inactivity, depression, smoking and low education levels, could result in an 8.3% reduction in the prevalence of Alzheimer's Disease (AD) by 2050 [1]. Given that cardiovascular risk factors often co-occur, several models have been developed to predict an individuals' risk of future cardiovascular disease (CVD) or stroke based on combinations of risk factors [2]. The most widely used in research and clinical settings are the Framingham risk models for predicting 10-year incident stroke, coronary heart disease (CHD) and general CVD [3]. These models usually incorporate cholesterol and blood pressure (BP) with a number of different additional variables such as age, smoking status, and ECG measures of heart health. These models generally have reasonable predictive accuracy for CVD events [2].

In older populations health-related co-morbidities are highly prevalent. Therefore, the investigation of the effect of multiple cardiovascular and health-related risk factors on cognitive function may be more relevant to real populations rather than examining individual risk factors in isolation. Further, the association between individual cardiovascular risk factors and cognitive function has been extensively studied [4]–[6]. With regards to combinations of risk factors, several population-based longitudinal studies have found different cardiovascular risk models to be associated with cognitive decline, including measures of global and domain specific (e.g., memory and non-memory) cognitive function [7]–[9]. For the purpose of specifically predicting later-life dementia, the Cardiovascular Risk Factors, Aging and Dementia (CAIDE) model, incorporating demographic variables (e.g., age, sex, education), midlife CVD risk factors (e.g., total cholesterol, systolic BP, body-mass index (BMI), physical activity) and/or Apolipoprotein E4 (ApoE4) status, was developed. This model has reasonable accuracy for predicting dementia [10], [11]. Given that numerous different CVD risk prediction models exist and a number of these have been tested within a dementia framework, evidence on comparisons is needed.

Accordingly, the aim of this systematic review was to synthesise evidence, from population-based studies, on the link between CVD risk prediction models on one hand, and longitudinal changes in cognition (e.g., global and domain specific) and risk of incident dementia on the other hand. Knowledge of the association of CVD risk models with cognitive outcomes is important for identifying individuals at high-risk of dementia whose risk could possibly be mitigated through management of risk factors or primary prevention strategies focused on CVD.

## Materials and Methods

### Selection criteria and search strategy

This review was undertaken in accordance with the PRISMA statement [12]. Medline, PsychINFO and Embase were searched from inception until March 28, 2014. Combinations of the following terms were searched: “cognit*”, “dementia”, “Alzheimer”, “Framingham”, “QRISK”, “CAIDE”, “ASSIGN”, “vascular risk”, “cardiovascular risk”, “stroke risk” and “cardiovascular health”. The search was restricted to articles published in English. One author completed the electronic search (SLH). Articles were included into the review based on the following criteria: 1) examined cardiovascular or stroke risk assessment models; 2) details of cognitive test scores (or change scores) were available at two or more time points or dementia incidence over time was reported; and, 3) longitudinal population-based study design. Articles were excluded if they only looked at individual risk factors, had a cross-sectional study design, or if they included cognitive test results in the risk model. Studies were not excluded based on the type of cardiovascular risk model or the neuropsychological test battery used to assess cognitive function. There was no restriction on sample age. Two authors (SLH and JD) first independently searched the article titles and abstracts. When a title/abstract of a study could not be rejected with certainty, the full text of the article was obtained for further investigation. The full text articles were then retrieved and assessed for eligibility. Relevant reviews were also retained and the reference lists of these and each included paper were reviewed for any missed articles. Any discrepancies between the selections made were resolved by consensus or by asking a third investigator (BCMS).

### Data extraction

Data were independently extracted by two authors (SLH and EYHT) including: date of publication, participants (country, follow-up assessment time), demographics (age and gender distributions), details of the risk model used and any modifications made, the main outcome measure (e.g., neuropsychological test or dementia incidence), analytical strategy (including multivariate adjustments) and (where reported) details on model prognostic performance including sensitivity, specificity, Area Under the Curve (AUC) or c-statistic. Discrepancies between data extraction results were resolved by reviewing the discrepancy. One investigator (SLH) assessed the quality of the included studies using the Newcastle-Ottawa Scale (NOS) for quality assessment of non-randomized studies, specifically cohort studies [13], as endorsed by the Cochrane collaboration [14]. The NOS uses a star rating system to assess selection, comparability and outcome criteria, with a maximum possible score of nine stars for each study.

## Results

### Main Search

**Figure 1:**
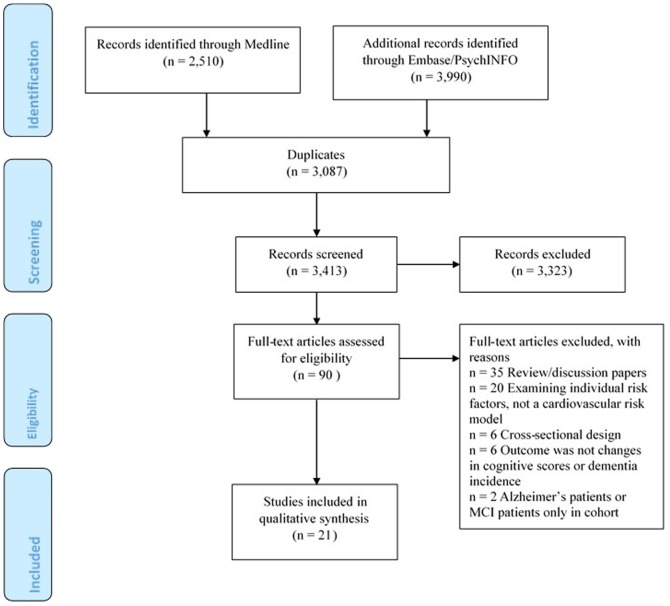
PRISMA flow chart diagram of the literature search.

The search identified 6,500 publications, of which 3,087 were duplicates and therefore removed. After reviewing titles/abstracts for eligibility, 90 publications were retained for full-text review. In total, 21 publications met the inclusion criteria and were retained. The main reasons for exclusion were that the articles were reviews or they examined individual cardiovascular risk factors, rather than using a cardiovascular disease risk model (Figure 1). It was not possible to conduct a meta-analysis due to the variability between studies including differences in inclusion of variables within the same risk models, limited number of studies using the same models, differences in cognitive outcomes, categorisation of the risk models (i.e., categorical or continuous) and differences in reporting the effect sizes.

### Study Characteristics

Table 1 shows a summary of the included studies and Tables S1, S2 and S3 show further details (online only). Three of the included studies were conducted with the Whitehall II study population [7], [9], [15], and two used the Reasons for Geographic and Racial Differences in Stroke (REGARDS) study data [16], [17]. Mean age at baseline ranged from 42.0 to 81.5 years, although three studies did not report the mean age for all participants [18]–[20]. Length of follow-up ranged from 2 to 36 years. The Canadian Study of Health and Aging had the smallest follow-up sample size of 223 participants [19], whereas, the REGARDS study had the largest follow-up sample size of >23,000 participants [16], [17].

**Table 1:** Summary of the articles included in the review.

Author	Follow- up sample (sex)	Outcome	Follow-up (yrs)	Baseline age (yrs)	CV risk model	Quality (Newcastle-Ottawa Scale)
**Studies examining the Framingham risk models**						
Brady (2001)	235 (all men)	Change in cognition scores	3	Mean = 66.4 (SD = 6.7)	Modified FSRP (omitted age)	*****
Dregan (2013)	8780 (men 3951, women 4829)	Change in cognition scores	4	50+ Mean = 62.5	Modified FSRP (LVH excluded) and Framingham CVD model	*******
Kaffashian (2011)	4827 (men 3486, women 1341)	Change in cognition scores	10	Mean = 55 (SD = 6) Range = 35 to 55	Framingham CVD model	*******
Kaffashian (2013)	CVD and CAIDE risk model n = 4374 (men 3162, women 1212) FSRP and CAIDE risk model n = 5157 (men 3651, women 1506)	Change in cognition scores	10	Mean = 55.6 Range = 35 to 55	Framingham CVD model, FSRP and CAIDE models 1 and 2	******
Kaffashian (2013)	5810 (men 4153, women 1657)	Changes in cognition scores	10	Mean = 55.6 Range = 35 to 55	FSRP	*******
Kelley (2013)	23830	Incident cognitive impairment	4	45+ Mean = 64.2	FSRP	******
Laughlin (2011)	985 (men 394, women 591)	Changes in cognition scores	Median = 9.8 (IQR = 2.5, 17.4)	Mean = 66.8 (SD = 8.5)	Framingham CHD model	*******
Unverzagt (2011)	23752	Incident cognitive impairment	Mean = 4.1	45+ Mean = 64.3	FSRP	******
Zeki Al Hazzouri (2013)	1116 (men 453, women 663)	Dementia and CIND incidence and change in cognition scores	10	Mean = 70 Range = 60 to 101	Framingham CVD model	*******
**Studies examining the CAIDE risk models**						
Exalto (2013)	9480	Dementia incidence	Mean = 36.1	Mean (SD) = 46.1 (4.3) Range = 40 to 55	CAIDE Model 1 plus additional risk factors	******
Kivipelto (2006)	1409 (men 534, women 875)	Dementia incidence	20	Mean (SD) = 50.4 (6.0) Range = 39 to 64	CAIDE Model 1 and CAIDE Model 2	******
Reijmer (2011)	322	Cognitive impairment	15	Mean (SD) = 55.9 (3.7) Range = 50 to 64	CAIDE Model 1	********
Reitz (2010)	1051	AD incidence	Mean (SD) = 4.0 (1.4)	65+ Mean (SD) = 75.7 (6.3)	Modified CAIDE Model 2.	********
Virta (2013)	2165 (men 1107, women 1058)	Moderate to severe cognitive impairment	Mean (SD) = 22.6 (2.3)	65+ Mean (SD) = 51.7 (6.1)	Modified CAIDE Model 1 and CAIDE Model 2	******
**Studies examining other cardiovascular risk models**						
Carmasin (2014)	435 (men 106, women 329)	Change in cognitive test scores	2.5	Mean (SD) = 66.8 (9.0)	Vascular risk factor index	****
Elkins (2004)	3832	Changes in cognitive test scores	5	65+	CHS Stroke risk model	********
Klages (2005)	223	AD or VCI incidence	5	65+	Vascularity Index model	********
Mitniski (2006)	380	Dementia incidence	20	75	Vascular index	******
Qiu (2010)	1270 (men 316, women 954)	Dementia incidence	Mean: 5.1	75+ Mean (SD) = 81.5 (5.0)	Vascular risk profile, Atherosclerotic risk profile and Hypoperfusion risk profile	********
Reis (2013)	2932 (men 1308, women 1624)	Changes in cognition scores	25	Range = 18 to 30	Ideal cardiovascular metrics	********
Whitmer (2005)	8845 (men 4094, women 4751)	Dementia incidence	Mean = 26.7	Mean = 42	Composite score based on 4 variables	********

The Newcastle-Ottawa Scale assesses selection, comparability and outcome criteria, with a maximum possible score of nine stars for each study. Abbreviations: AD, Alzheimer's disease; CAIDE, Cardiovascular Risk Factors, Aging and Dementia; CIND, cognitive impairment no dementia; CHD, coronary heart disease; CHS, Cardiovascular Health Study; CVD, cardiovascular disease; FSRP, Framingham Stroke Risk Profile; IQR, inter-quartile range; LVH, left-ventricular hypertrophy; SD, standard deviation; VCI, vascular cognitive impairment.

### Quality of the included studies

Articles were assessed on selection, comparability and outcome. The majority of studies showed a low risk of selection bias (n = 19 used clinical examinations and medical records to ascertain the variables required for the cardiovascular risk models, and only two studies based this on self-report). Most studies (n = 20) were defined as representative of the general older population, but one study [21] demonstrated bias as it only included males. Adjustment for confounding factors varied widely. All studies used an established cognitive test or validated dementia criteria as the outcome. However, many different cognitive tests were used raising issues of comparability across the studies. Loss to follow-up was high; 20 studies had a follow-up rate <80% or made no statement about loss to follow-up.

### The different risk models and dementia

Seven studies examined seven different risk models with dementia as the outcome including: the Framingham CVD model [22], the CAIDE model (three studies: one study used CAIDE model 1 [11], one study used CAIDE model 2 (with ApoE4 status included) [23] and one study examined both CAIDE models [10]), the vascularity index model [19], the vascular index [24], the atherosclerotic risk profile [25], the hypoperfusion risk profile [25] and a cardiovascular composite model [26]. In one study the outcome was incident AD [19] and in the remaining studies the outcome was incident dementia (all-cause) [10], [11], [23]–[26].

All studies, except one [19], found the different risk models to be significantly associated with an increased risk of future dementia. In the study that showed non-significant results, a vascular index incorporating history of stroke, transient ischaemic attack, heart attack, diabetes mellitus, smoking, use of antihypertensive medications and systolic BP was not associated with risk of AD over five years in a population-based sample aged 65 years and older. However, the vascular index model was found to be associated with an increased risk of vascular cognitive impairment (VCI) [19].

Where model prognostic performance indices were reported findings were mixed [10], [11], [19], [24]. Two studies reported moderate prognostic performance for the CAIDE models dementia (AUC 0.77 (model 1) and 0.78 (model 2) over 20 years follow-up [10] and 0.75 (model 1) over 36 years follow-up [11]). One study added additional risk factors to CAIDE model 1 including central obesity, depressed mood, diabetes mellitus, head trauma, poor lung function and smoking, but these variables did not significantly improve predictive accuracy [11]. The vascular index model, incorporating history of stroke, transient ischaemic stack, heart attack and diabetes, smoking, use of hypertensive medications and BP, predicted incident dementia with different accuracy depending on follow-up time (AUC 0.67 for 20 years and 0.74 for 10 years) [19]. The vascular index described in the Gothenburg H-70 cohort that incorporates fourteen different variables including hypertension, BMI, cholesterol, dizziness, calf pain, chest pain, second heart sound abnormal, aortic calcification, pulmonary congestion, t-wave abnormalities, atrial fibrillation, sinus tachycardia, diabetes and angina pectoris had low discriminative accuracy for predicting dementia over 20 years (AUC 0.67) [24]. The remaining studies where dementia was an outcome did not report prognostic performance indices [22], [23], [25], [26].

### The Framingham cardiovascular risk models and cognitive decline

Four studies used the Framingham CVD model [7], [8], [15], [22], six studies used the Framingham stroke model [7]–[9], [16], [17], [21] and one study used the Framingham CHD model [27]. Calculation of the Framingham stroke risk score was slightly modified in two studies; one omitted age [21] and the other omitted left ventricular hypertrophy (LVH) [8]. All studies found higher Framingham risk scores (i.e., CVD, CHD and stroke) to be associated with an increased risk of future cognitive decline [7]–[9], [15], [21], [22], [27] or cognitive impairment [16], [17], [22]. Studies differed as to which cognitive abilities were associated with cardiovascular or stroke risk. While some studies found significant associations across all cognitive domains tested, others found the association to be present only for certain cognitive domains including verbal fluency [21] and reasoning [15]. When stratified by sex, two studies found sex-specific associations (women [27] or men [15] only), although the association only in women may have been due to selective attrition of men with greater levels of cognitive decline [27].

### The CAIDE models and cognitive decline

Two studies examining the CAIDE models had incident cognitive impairment as an outcome [28], [29] and one study investigated changes in cognitive test scores [7]. One study only examined CAIDE model 1 (without ApoE4 status included) [28] and two studies examined both CAIDE models [7], [29]. The CAIDE models (both models 1 and 2) were found to be associated with cognitive decline [7] and incident cognitive impairment [28], [29]. Where specific domains were investigated, the CAIDE models predicted changes in reasoning, memory, vocabulary, information processing speed, visuo-construction and abstract reasoning, but not, language or attention and executive functioning [28], attention, memory, orientation or abstraction [29]. One study that compared the CAIDE models with the Framingham CVD model and the Framingham stroke risk model concluded that all of the models predicted cognitive decline, but the Framingham models were significantly better than the CAIDE models for predicting changes in semantic fluency and global cognition [7]. One study examined the prognostic performance of the CAIDE models in relation to incident cognitive impairment and found moderate predictive accuracy (CAIDE model 1 AUC 0.74 and CAIDE model 2 AUC 0.75) [29].

### Other cardiovascular risk models and cognitive decline

Seven studies adopted nine other cardiovascular risk prediction models [18]–[20], [24]–[26], [30] (these include those examining both cognitive changes and dementia incidence as outcomes). All of these risk models included a measure of BP and eight included a measure of glucose level or diabetes status as well as other variables such as physical activity, cholesterol levels and a history of CVD (Table 2). The number of variables incorporated into the cardiovascular risk models ranged from three to fifteen. All models examined showed significant positive associations with future cognitive decline including measures of processing speed, psychomotor speed, executive function and verbal memory [18], [20], [30].

**Table 2:** The different cardiovascular risk models and the variables included.

Cardiovascular risk model	Variables included
Framingham cardiovascular risk models	
Framingham CVD model	Age, total cholesterol, HDL cholesterol, systolic blood pressure if not treated, systolic blood pressure if treated, current smoker and diabetes.
FSRP	Age, systolic blood pressure, antihypertensive therapy, current smoker, diabetes, CVD, atrial fibrillation and LVH.
Framingham CHD model	Age, total cholesterol or LDL cholesterol, HDL cholesterol, systolic and diastolic blood pressure, current smoker and diabetes.
CAIDE risk models	
CAIDE Model 1	Age, total cholesterol, systolic blood pressure, BMI, sex, education and physical activity.
CAIDE Model 2	Age, total cholesterol, systolic blood pressure, BMI, sex, education, physical activity and ApoE4 status.
Other cardiovascular risk models	
Vascular risk factor index	Diabetes, CVD, blood pressure, history of a heart attack, angina, circulation problems and history of stroke.
CHS Stroke risk model	Age, systolic blood pressure, diabetes mellitus, impaired fasting glucose, atrial fibrillation, LVH, sex, history of heart disease, creatinine level and 15ft walk time.
Vascularity index model	History of stroke, transient ischaemic attack, heart attack, diabetes, smoking, use of antihypertensive medications and sitting systolic blood pressure.
Vascular index	Dizziness, calf pain, chest pain, second heart sound abnormal, aortic calcification, pulmonary congestion, t-wave abnormalities, atrial fibrillation, sinus tachycardia, diabetes, hypertension, treated hypertension, angina pectoris, BMI, cholesterol.
Vascular risk profile	Systolic blood pressure, diastolic blood pressure, pulse pressure, diabetes/pre-diabetes, stroke and heart failure.
Atherosclerotic risk profile	Systolic blood pressure, diabetes/pre-diabetes and stroke.
Hypoperfusion risk profile	Diastolic blood pressure, pulse pressure and heart failure.
Ideal cardiovascular metrics	BMI, diet score, smoking, physical activity, total cholesterol, blood pressure and fasting glucose.
Composite vascular score	Hypertension, smoking, cholesterol and diabetes.

Abbreviations: ApoE, Apolipoprotein E; BMI, body mass index; CAIDE, Cardiovascular Risk Factors, Aging and Dementia, CHD, coronary heart disease; CHS, cardiovascular health study; CVD, cardiovascular disease; FSRP, Framingham stroke risk profile; HDL, high-density lipoprotein; LDL, low-density lipoprotein; LVH, left ventricular hypertrophy.

## Discussion

This systematic review of longitudinal population-based studies found that a wide-variety of CVD risk prediction models have been associated with cognitive decline or future risk of cognitive impairment or dementia. There was however, large variability in studies with regard to the cognitive domains tested and the pattern of associations. Some studies observed differences across all cognitive domains including memory, non-memory and global cognitive function, whilst others only observed significant associations for specific cognitive domains such as reasoning and vocabulary. In the few studies that tested prognostic performance [10], [11], [24], [29] predictive accuracy for incident dementia were found to be moderate (AUC range 0.74 to 0.78) across different CVD risk prediction models.

The main strength of this study is that it is the first systematic review undertaken to summarise current evidence on the association between CVD risk predictions models and cognitive decline and dementia. The review is wide in scope and included studies that examined dementia incidence as well as changes in cognitive test scores as outcomes over time. There are some limitations. Only studies published in English were included. Also, the included studies were characterised by large variability in study design (e.g., follow-up time, sample size) as well as the risk model evaluated (and modifications made to calculating the risk score) and the outcome measures used. Therefore it was not possible to synthesise the results into a meta-analysis. In order to compare studies, better consistency of methodological approaches is needed.

Each model in this review included different risk factors selected to accurately predict the outcome they were originally designed for including: CVD, stroke, CHD, overall cardiovascular health, AD, depression and all-cause dementia. Apart from the CAIDE, none of the vascular index, the composite vascular model and the vascularity index models, were designed to predict changes in cognitive function or incident dementia. What risk factors should be included to predict future cognitive decline and dementia requires the rationale for their inclusion in a risk score as well as further empirical studies testing the best combination of predictors from a wide-range of well-justified risk factors for different outcomes. The results here suggest that the Framingham models could be a good starting point for future validation analysis.

All CVD risk models included BP and most included cholesterol, diabetes mellitus and smoking status. Each of these factors has been associated with cognitive decline and dementia [6]. However, CVD and its risk factors rarely occur in isolation, particularly in older aged individuals where disease related co-morbidity and multi-morbidity is high [31], [32]. In addition, an advantage of such CVD risk models is that they are typically calculated in clinical settings and could be used not only to ascertain an individual's vascular health status, but as suggested by the results of this review, may also be useful to inform clinicians of the individual's risk of future cognitive decline and dementia. Clinicians may then able to adopt similar CVD risk reducing strategies that will not only benefit cardiovascular health, but also future cognitive wellbeing. The advantage of this is that although clinicians may not be acutely aware of cognitive intervention strategies, they are well versed in cardiovascular health risk reduction strategies e.g., reducing smoking, alcohol and BP management.

Across the different CVD risk models associations with cognitive function varied depending on the risk score calculated, outcome tested and participant gender. Generally the different CVD risk models were associated with at least one cognitive measure over time or incident dementia. Significant associations were found between CVD risk models and specific cognitive measures for global, memory and non-memory measures including category fluency, executive functioning, reasoning, phonemic fluency, semantic fluency, vocabulary, information processing speed and visuo-construction performance. Yet, there was variability between studies in terms of associations with specific domain measure e.g., the English Longitudinal Study of Ageing found a significant association between the Framingham stroke risk model and the Framingham CVD model and memory decline [8], whereas, the Whitehall II Study did not find a significant association between the Framingham risk models and changes in memory [7],[9],[15]. Further, a comparative study of the CAIDE models with the Framingham stroke risk model and Framingham CVD models found a stronger association between changes in global cognition and semantic fluency and Framingham models compared to the CAIDE models [7]. The study suggested that as the CAIDE models include education they may be better suited to its' original purpose for detecting dementia risk rather than predicting cognitive decline, as education was not found to be associated with 10-year cognitive decline in the original study. The combination of cardiovascular risk variables in the Framingham models may have led to the greater association with cognitive decline [7].

The studies examined in this review had very different follow-up times (range: 2.5 [30] to 36.1 years [11]). This shows that there is potentially a long time to intervene and target vascular risk factors to reduce risk of future cognitive decline or dementia. Further, it has been well established that vascular risk factors have different magnitude and even direction of associations with dementia risk depending on whether they are examined at mid or later-life. For instance, high BP has been found to be positively associated with cognitive decline when measured at mid-life, but has been found to have a potentially beneficial effect towards cognitive decline in the oldest-old, those aged 85 years and older [33]. However, it is uncertain whether this is reverse causation as BP may be lower in those who experience greater cognitive decline due to underlying dementia pathology [34]. In this review, no study examined the association between cardiovascular risk and cognitive changes in the oldest-old, but cardiovascular risk models were found to be associated with cognitive decline in the older population (four studies were conducted in populations with a mean age 70 years and older). This suggests that determining cardiovascular risk even in later-life may be useful for understanding cognitive decline or dementia risk.

Only four studies have calculated and compared model prognostic performance; three studies tested this for the CAIDE models [10], [11], [29] and one study tested this for a non-Framingham vascular risk model [24]. The results were mixed. The CAIDE model was found to have higher accuracy for predicting dementia compared to a vascular index model [11], [24]. The vascular index and the CAIDE models only share three variables including: systolic BP, BMI and cholesterol. The vascular index contains additional variables related to cardiovascular history and history of other diseases (e.g., angina pectoris and diabetes); whereas, the CAIDE models contain additional variables related to demographic, lifestyle and genetic factors (age, sex, education, physical activity and ApoE4). These results suggest that a combination of both cardiovascular, lifestyle and genetic factors may potentially be useful for dementia risk prediction.

In contrast, the predictive accuracy of the Framingham models has not been examined in relation to incident dementia or cognitive impairment. Therefore, it remains unknown how predictive the Framingham models are for cognitive changes and dementia over time. Further research is needed to compare the predictive accuracy of the Framingham and non-Framingham models. This is especially important; as compared to other dementia risk prediction models the Framingham models contain variables that are already easily collectable within clinical practice (for a systematic review of dementia specific risk models see [35]).

## Conclusion

Overall, a strong positive association was observed between the different vascular risk models and future cognitive decline or incident cognitive impairment or dementia. Importantly, such scores are easily obtainable in clinical and research settings and may be useful for identifying those individuals in a population who are at highest risk of future cognitive decline and dementia. However, before recommendations can be made as to which model works best and whether any can be recommended for use within a cognitive/dementia risk prediction framework further validation and comparative work is needed. This is particularly important with regard to testing model prognostic performance in different samples (e.g., population-based vs. clinical) with different ethnic, health and socio-demographic characteristics (e.g., age and sex).

## Supporting Information

Table S1 Summary of articles with dementia as an outcome.
(DOC)

Table S2 Summary of articles with dementia and cognitive changes as outcomes.
(DOC)

Table S3 Summary of articles with cognitive changes as the outcome.
(DOC)

Materials S1 **Sample search strategy.**
(DOCX)

Checklist S1 **PRISMA checklist.**
(DOC)
