# Supplementary material for: Cardiovascular Disease Risk Models and Longitudinal Changes in Cognition: A Systematic Review
Source: PLoS One. 2014 Dec 5;9(12):e114431. doi: 10.1371/journal.pone.0114431 (PMC4257686; doi:10.1371/journal.pone.0114431)
Supplement: Table S1 — Summary of articles with dementia as an outcome. (DOC) [file pone.0114431.s001.doc]

|  | **Supplementary Table 1 Summary of articles with dementia as an outcome** | | | | | | | | | | | |
| --- | --- | --- | --- | --- | --- | --- | --- | --- | --- | --- | --- | --- |
| **Sample** | | **Follow- up sample (sex)** | **Outcome** | **Follow-up (years)** | **Baseline age (years)** | **CV risk score** | **Cognitive assessments or dementia criteria** | **Outcome measure** | **Results** | **Multivariate adjustment** | **General conclusion** |  |
| CAIDE models | | | | |  |  |  |  |  |  |  |  |
| Kaiser PermanentMedical Care Program of Northern California (KPNC) | | 9480 | Dementia incidence | Mean: 36.1 | 40-55; Mean 46.1 (sd: 4.3) | CAIDE Model 1 plus additional risk factors | ICD-9 | c-statistic | 2767 (25%) any type of dementia 1011 (10.7%) specialist-confirmed diagnosis | None | The CAIDE risk score is predictive of the likelihood of developing dementia later in life, adding other midlife risk factors did not improve predictability. |  |
|  | |  |  |  |  |  |  |  | c-statistic (logistic) |  |  |  |
|  | |  |  |  |  |  |  |  | CAIDE 0.747 |  |  |  |
|  | |  |  |  |  |  |  |  | CAIDE (Asian) 0.812 |  |  |  |
|  | |  |  |  |  |  |  |  | CAIDE (Black) 0.750 |  |  |  |
|  | |  |  |  |  |  |  |  | CAIDE (White) 0.735 |  |  |  |
|  | |  |  |  |  |  |  |  | CAIDE + central obesity 0.747 |  |  |  |
|  | |  |  |  |  |  |  |  | CAIDE + depressed mood 0.747 |  |  |  |
|  | |  |  |  |  |  |  |  | CAIDE + head trauma 0.747 |  |  |  |
|  | |  |  |  |  |  |  |  | CAIDE + poor lung function 0.747 |  |  |  |
|  | |  |  |  |  |  |  |  | CAIDE + smoking 0.749 |  |  |  |
|  | |  |  |  |  |  |  |  | c-statistic (cox) |  |  |  |
|  | |  |  |  |  |  |  |  | CAIDE 0.665 |  |  |  |
|  | |  |  |  |  |  |  |  | CAIDE + central obesity 0.668 |  |  |  |
|  | |  |  |  |  |  |  |  | CAIDE + depressed mood 0.665 |  |  |  |
|  | |  |  |  |  |  |  |  | CAIDE + diabetes mellitus 0.665 |  |  |  |
|  | |  |  |  |  |  |  |  | CAIDE + head trauma 0.665 |  |  |  |
|  | |  |  |  |  |  |  |  | CAIDE + poor lung function 0.666 |  |  |  |
|  | |  |  |  |  |  |  |  | CAIDE + smoking 0.665 |  |  |  |
| Cardiovascular Risk Factors, Aging, and Dementia (CAIDE) study | | 1409 (men 534, women 875) | Dementia incidence | 20 | 39-64; Mean 50.4 (sd: 6.0) | CAIDE Model 1 and CAIDE Model 2 | DSM-IV | AUC | Dementia incidence: 61 | None | The CAIDE score predicts future dementia well; however, the CAIDE score needs to be validated in another population. |  |
|  | |  |  |  |  |  |  |  | AUC (95% CI) |  |  |  |
|  | |  |  |  |  |  |  |  | CAIDE Model 1: 0.77 (0.71, 0.83) |  |  |  |
|  | |  |  |  |  |  |  |  | CAIDE Model 2: 0.78 (0.72, 0.84) |  |  |  |
|  | |  |  |  |  |  |  |  | CAIDE Model 1 cut off score 9: sensitivity 0.77, specificity 0.63; cut off score 10: sensitivity 0.63, specificity 0.75 |  |  |  |
|  | |  |  |  |  |  |  |  | CAIDE Model 2 cut off score 10: sensitivity 0.81, specificity 0.61; cut off score 11: sensitivity 0.67, specificity 0.72 |  |  |  |
| Other cardiovascular risk models | | | |  |  |  |  |  |  |  |  |  |
| Medicare recipients residing in northern Manhattan | | 1051 | AD incidence | Mean 4.0 (sd: 1.4) | Mean 75.7 (sd: 6.3) | Modified CAIDE Model 2 | DSM-IV. AD by NINCDS-ADRDA. | HR | 92 incident probable and possible AD 80 incident probable AD | Age, sex, education, and ethnicity | This vascular risk score could be a valuable tool to identify elderly individuals who might be at increased risk of AD. |  |
|  | |  |  |  |  |  |  |  | Probable and possible AD, Risk score, HR (95% CI) |  |  |  |
|  | |  |  |  |  |  |  |  | 0-14, 1 (ref) |  |  |  |
|  | |  |  |  |  |  |  |  | 15–18, 3.74 (1.42, 9.88) p** |  |  |  |
|  | |  |  |  |  |  |  |  | 19–22, 3.55 (1.31, 9.62) p** |  |  |  |
|  | |  |  |  |  |  |  |  | 23–28, 12.57 (5.26, 30.08) p*** |  |  |  |
|  | |  |  |  |  |  |  |  | >28, 20.47 (8.38, 49.99) p*** |  |  |  |
|  | |  |  |  |  |  |  |  | >28, 20.47 (8.38, 49.99) p*** |  |  |  |
|  | |  |  |  |  |  |  |  | P value for trend*** |  |  |  |
|  | |  |  |  |  |  |  |  | Probable AD, Risk Score, HR (95% CI) |  |  |  |
|  | |  |  |  |  |  |  |  | 0–16, 1 (ref) |  |  |  |
|  | |  |  |  |  |  |  |  | 17–21, 3.381 (0.87, 13.13) p ns |  |  |  |
|  | |  |  |  |  |  |  |  | 22–26, 4.98 (1.36, 18.19) p** |  |  |  |
|  | |  |  |  |  |  |  |  | 27–32, 14.01 (4.06, 48.39) p*** |  |  |  |
|  | |  |  |  |  |  |  |  | >32, 35.81 (10.81, 118.61) p*** |  |  |  |
|  | |  |  |  |  |  |  |  | P value for trend*** |  |  |  |
| Gothenburg H-70 1901-02 Birth Cohort | | 380 | Dementia incidence | 20 | 75 | Vascular index | DSM-III-R | AUC | Dementia incidence: 103 AUC=0.74 (SE=0.02) for 10 years AUC=0.67 (SE=0.02) for 20 years | None | Vascular risk factor indexes give robust estimates for dementia in elderly people. |  |
| Kungsholem Project | | 1270 (men 316, women 954) | Dementia incidence | Mean: 5.1 | 75+ Mean (sd): 81.5 (5.0) | Vascular risk profile, Atherosclerotic risk profile and Hypoperfusion risk profile | DSM-III-R | HR | Dementia incidence: 428 (328 AD) | Age, gender, education, ApoE status, follow-up survival bias, baseline MMSE score, BMI, CHD, use of bp lowering drugs, and if applicable for atherosclerotic or hypoperfusion profile. | Aggregation of atherosclerotic and hypoperfusion-related vascular factors increases the risk of dementia in the very old. |  |
|  | |  |  |  |  |  |  |  | Vascular risk profile and dementia HR (95% CI) |  |  |  |
|  | |  |  |  |  |  |  |  | 0=ref (1.00) |  |  |  |
|  | |  |  |  |  |  |  |  | 1=1.11 (0.79, 1.58) |  |  |  |
|  | |  |  |  |  |  |  |  | 2=1.65 (1.12, 2.42) |  |  |  |
|  | |  |  |  |  |  |  |  | 3+=2.48 (1.46, 4.20) |  |  |  |
|  | |  |  |  |  |  |  |  | p for trend*** |  |  |  |
|  | |  |  |  |  |  |  |  | Vascular risk profile and AD HR (95% CI) |  |  |  |
|  | |  |  |  |  |  |  |  | 0=ref (1.00) |  |  |  |
|  | |  |  |  |  |  |  |  | 1=1.09 (0.75, 1.60) |  |  |  |
|  | |  |  |  |  |  |  |  | 2=1.77 (1.16, 2.71) |  |  |  |
|  | |  |  |  |  |  |  |  | 3+=2.66 (1.39, 5.08) |  |  |  |
|  | |  |  |  |  |  |  |  | p for trend*** |  |  |  |
|  | |  |  |  |  |  |  |  | Atherosclerotic risk profile and dementia HR (95% CI) |  |  |  |
|  | |  |  |  |  |  |  |  | 0=ref (1.00) |  |  |  |
|  | |  |  |  |  |  |  |  | 1=1.28 (1.00, 1.64) |  |  |  |
|  | |  |  |  |  |  |  |  | 2+=2.13 (1.46, 3.11) |  |  |  |
|  | |  |  |  |  |  |  |  | p for trend*** |  |  |  |
|  | |  |  |  |  |  |  |  | Atherosclerotic risk profile and AD HR (95% CI) |  |  |  |
|  | |  |  |  |  |  |  |  | 0=ref (1.00) |  |  |  |
|  | |  |  |  |  |  |  |  | 1=1.33 (1.00, 1.78) |  |  |  |
|  | |  |  |  |  |  |  |  | 2+=2.09 (1.31, 3.34) |  |  |  |
|  | |  |  |  |  |  |  |  | p for trend*** |  |  |  |
|  | |  |  |  |  |  |  |  | Hypoperfusion risk profile and dementia HR (95% CI) |  |  |  |
|  | |  |  |  |  |  |  |  | 0=ref (1.00) |  |  |  |
|  | |  |  |  |  |  |  |  | 1=1.15 (0.90, 1.47) |  |  |  |
|  | |  |  |  |  |  |  |  | 2+=1.88 (1.30, 2.70) |  |  |  |
|  | |  |  |  |  |  |  |  | p for trend*** |  |  |  |
|  | |  |  |  |  |  |  |  | Hypoperfusion risk profile and AD HR (95% CI) |  |  |  |
|  | |  |  |  |  |  |  |  | 0=ref (1.00) |  |  |  |
|  | |  |  |  |  |  |  |  | 1=1.27 (0.96, 1.69) |  |  |  |
|  | |  |  |  |  |  |  |  | 2+=2.06 (1.35, 3.13) |  |  |  |
|  | |  |  |  |  |  |  |  | p for trend*** |  |  |  |
| Kaiser Permanent Medical Care Program of Northern California (KPNC) | | 8845 (men 4094, women 4751) | Dementia incidence | Mean: 26.7 | 40 – 44 Mean: 42 | Composite score based on 4 variables (Smoking, hypertension, high cholesterol, and diabetes) | ICD-9-CM and CPT4 | HR | Dementia incidence: 721 | Age at midlife exam, age at start of case ascertainment, race, education and sex. | Cardiovascular composite score was associated with risk of late-life dementia in a dose dependent fashion. |  |
|  | |  |  |  |  |  |  |  | HR (95% CI) |  |  |  |
|  | |  |  |  |  |  |  |  | 1=1.27 (1.02, 1.58) |  |  |  |
|  | |  |  |  |  |  |  |  | 2=1.69 (1.34, 2.12) |  |  |  |
|  | |  |  |  |  |  |  |  | 3=2.31 (1.71, 3.11) |  |  |  |
|  | |  |  |  |  |  |  |  | 4=2.37 (1.10, 5.10) |  |  |  |

Abbreviations: 3MSE, Modified Mini-Mental State Exam; AD, Alzheimer’s disease; bp, blood pressure; ApoE, Apolipoprotein E; AUC, area under the curve; BMI, body mass index; CI, confidence interval; BCRT, Buschke Cued Recall Test; CHD, coronary heart disease; CHS, Cardiovascular Health Study; DSM-III-R, Diagnostic and Statistical Manual for Mental Disorders-III-revised; DSST, Digit Symbol Substitution Test; HR, Hazard Ratio; ICD-9-CM, International Classification of Diseases, Ninth Revision, Clinical Modification; ICD-10, International Classification of disease version 10; LVH, left-ventricular hypertrophy; MMSE, Mini-Mental State Examination; MRI, magnetic resonance imaging; NINCDS-ADRDA, National Institute of Neurological and Communicative Disorders and Stroke-Alzheimer Disease and Related Disorders Association; OR, Odds Ratio; RAVLT, Rey Auditory Verbal Learning Test; ref, reference category; sd, standard deviation; TIA, transient ischaemic attack; VaD, Vascular dementia; VCI, vascular cognitive impairment; WAIS, Wechsler Adult Intelligence Scale.

* p<0.05 **p<0.01 ***p<0.001 ns=non-significant

**References**

1. Exalto LG, Quesenberry CP, Barnes D, Kivipelto M, Biessels GJ, et al. (2013) Midlife risk score for the prediction of dementia four decades later. Alzheimers & Dementia.

2. Kivipelto M, Ngandu T, Laatikainen T, Winblad B, Soininen H, et al. (2006) Risk score for the prediction of dementia risk in 20 years among middle aged people: a longitudinal, population-based study. Lancet Neurology 5: 735-741.

3. Reitz C, Tang MX, Schupf N, Manly JJ, Mayeux R, et al. (2010) A summary risk score for the prediction of Alzheimer disease in elderly persons. Arch Neurol 67: 835-841.

4. Mitnitski A, Skoog I, Song X, Waern M, Ostling S, et al. (2006) A vascular risk factor index in relation to mortality and incident dementia. European Journal of Neurology 13: 514-521.

5. Qiu C, Xu W, Winblad B, Fratiglioni L (2010) Vascular risk profiles for dementia and Alzheimer's disease in very old people: a population-based longitudinal study. Journal of Alzheimer's Disease 20: 293-300.

6. Whitmer RA, Sidney S, Selby J, Claiborne Johnston S, Yaffe K (2005) Midlife cardiovascular risk factors and risk of dementia in late life. Neurology 64: 277-281.
