# Supplementary material for: Cardiovascular Disease Risk Models and Longitudinal Changes in Cognition: A Systematic Review
Source: PLoS One. 2014 Dec 5;9(12):e114431. doi: 10.1371/journal.pone.0114431 (PMC4257686; doi:10.1371/journal.pone.0114431)
Supplement: Table S2 — Summary of articles with dementia and cognitive changes as outcomes. (DOC) [file pone.0114431.s002.doc]

|  | **Supplementary Table 2 Summary of articles with dementia and cognitive changes as outcomes** | | | | | | | | | | | |
| --- | --- | --- | --- | --- | --- | --- | --- | --- | --- | --- | --- | --- |
| **Sample** | | **Follow- up sample (sex)** | **Outcome** | **Follow-up (years)** | **Baseline age (years)** | **CV risk score** | **Cognitive assessments or dementia criteria** | **Outcome measure** | **Results** | **Multivariate adjustment** | **General conclusion** |  |
| Framingham models | | | |  |  |  |  |  |  |  |  |  |
| Sacramento Area Latino Study on Aging (SALSA) | | 1116 (men 453, women 663) | Dementia and CIND incidence and change in cognition scores | 10 | 60 to 101. Mean 70 | Framingham CVD Score (Percentiles) | 3MSE SEVLT Multistage screening process for diagnosing dementia; final stage involved DSM-IV and NINCDS-ADRDA | β coefficient for cognition scores and HR for dementia/CIND incidence | 3MSE, SEVLT and dementia/CIND incidence scores. | Age, education and nativity | Framingham CVD score was associated with cognitive decline and dementia/CIND incidence in elderly Mexican Americans. |  |
|  | |  |  |  |  |  |  |  | 3MSE β (95% CI) |  |  |  |
|  | |  |  |  |  |  |  |  | Women |  |  |  |
|  | |  |  |  |  |  |  |  | 25th Percentile -0.41 (-1.14, -0.31) |  |  |  |
|  | |  |  |  |  |  |  |  | 50th Percentile 0.11(-0.51, 0.72) |  |  |  |
|  | |  |  |  |  |  |  |  | 75th Percentile 0.83 (0.12, 1.53) |  |  |  |
|  | |  |  |  |  |  |  |  | Men |  |  |  |
|  | |  |  |  |  |  |  |  | 25th Percentile -1.76 (-2.78, -0.73) |  |  |  |
|  | |  |  |  |  |  |  |  | 50th Percentile -0.96 (-1.81, -0.10) |  |  |  |
|  | |  |  |  |  |  |  |  | 75th Percentile 0.12 (-0.82, 1.07) |  |  |  |
|  | |  |  |  |  |  |  |  | SEVLT β (95% CI) |  |  |  |
|  | |  |  |  |  |  |  |  | Women |  |  |  |
|  | |  |  |  |  |  |  |  | 25th Percentile -0.09 (-0.11, -0.06) |  |  |  |
|  | |  |  |  |  |  |  |  | 50th Percentile -0.10(-0.12, -0.08) |  |  |  |
|  | |  |  |  |  |  |  |  | 75th Percentile -0.12 (-0.14, -0.09) |  |  |  |
|  | |  |  |  |  |  |  |  | Men |  |  |  |
|  | |  |  |  |  |  |  |  | 25th Percentile -0.03 (-0.06, 0.01) |  |  |  |
|  | |  |  |  |  |  |  |  | 50th Percentile -0.04 (-0.07, -0.01) |  |  |  |
|  | |  |  |  |  |  |  |  | 75th Percentile -0.05 (-0.09, -0.02) |  |  |  |
|  | |  |  |  |  |  |  |  | Dementia/CIND incidence HR (95% CI) ref education<6 years |  |  |  |
|  | |  |  |  |  |  |  |  | Women education 6-11 years |  |  |  |
|  | |  |  |  |  |  |  |  | 25th Percentile 0.68 (0.28, 1.64) |  |  |  |
|  | |  |  |  |  |  |  |  | 50th Percentile 0.70 (0.35, 1.40) |  |  |  |
|  | |  |  |  |  |  |  |  | 75th Percentile 0.74 (0.42, 1.33) |  |  |  |
|  | |  |  |  |  |  |  |  | Women education >=12 years |  |  |  |
|  | |  |  |  |  |  |  |  | 25th Percentile 0.24 (0.08, 0.71) |  |  |  |
|  | |  |  |  |  |  |  |  | 50th Percentile 0.34 (0.15, 0.79) |  |  |  |
|  | |  |  |  |  |  |  |  | 75th Percentile 0.55 (0.28, 1.07) |  |  |  |
|  | |  |  |  |  |  |  |  | Men education 6-11 years |  |  |  |
|  | |  |  |  |  |  |  |  | 25th Percentile 0.69 (0.18, 2.65) |  |  |  |
|  | |  |  |  |  |  |  |  | 50th Percentile 0.75 (0.27, 2.11) |  |  |  |
|  | |  |  |  |  |  |  |  | 75th Percentile 0.83 (0.38, 1.82) |  |  |  |
|  | |  |  |  |  |  |  |  | Men education >=12 years |  |  |  |
|  | |  |  |  |  |  |  |  | 25th Percentile 0.90 (0.25, 3.25) |  |  |  |
|  | |  |  |  |  |  |  |  | 50th Percentile 0.75 (0.28, 2.02) |  |  |  |
|  | |  |  |  |  |  |  |  | 75th Percentile 0.59 (0.22, 1.61) |  |  |  |
| Other cardiovascular risk models | | | | | | | | | | | |  |
| Canadian Study of Health and Aging | | 223 | AD or VCI incidence | 5 | 65+ | Vascularity Index Score | DSM-III-R, NINCDS-ADRDA for AD, ICD-10 for VaD, staged process for VCI | OR | Incidence 20 AD and 21 VCI. Risk of AD OR (95% CI) 0.68 (0.38, 1.20) and Risk of VCI OR (95% CI)  2.18 (1.36, 3.51) | ApoE status, sex, age education and BCRT score | Vascularity Index increased the risk of VCI, but not AD. |  |

Abbreviations: AD, Alzheimer’s disease; AH4-I, Alice Heim 4-I; AUC, area under the curve; CAIDE, Cardiovascular Risk Factors, Aging and Dementia; CI, confidence interval; CVD, cardiovascular disease; DSM-IV, Diagnostic and Statistical Manual for Mental Disorders-IV; FSRP, Framingham Stroke Risk Profile; HDL, High-density lipoprotein; HR, hazard ratio; ICD-9, International Classification of disease version 9; NINCDS-ADRDA, National Institute of Neurological and Communicative Disorders and Stroke-Alzheimer Disease and Related Disorders Association; OR, odds ratio; ref, reference category; sd, standard deviation; RF, risk factor; TELE, validated telephone interview; WAIS-III, Wechsler Adult Intelligence Scale;

* p<0.05 **p<0.01 ***p<0.001 ns=non-significant

**References**

1. Zeki Al Hazzouri A, Haan M (2013) Cardiovascular Risk Score, Cognitive Decline and Dementia in Older Mexican Americans: The Role of Gender and Education. American Journal of Epidemiology 177: S3-S3.

2. Klages JD, Fisk JD, Rockwood K (2005) APOE genotype, vascular risk factors, memory test performance and the five-year risk of vascular cognitive impairment or Alzheimer's disease. Dementia & Geriatric Cognitive Disorders 20: 292-297.
