# Supplementary material for: Cardiovascular Disease Risk Models and Longitudinal Changes in Cognition: A Systematic Review
Source: PLoS One. 2014 Dec 5;9(12):e114431. doi: 10.1371/journal.pone.0114431 (PMC4257686; doi:10.1371/journal.pone.0114431)
Supplement: Table S3 — Summary of articles with cognitive changes as the outcome. (DOC) [file pone.0114431.s003.doc]

| **Supplementary Table 3 Summary of articles with cognitive changes as the outcome** | | | | | | | | | | | | | | | | | | | |  |
| --- | --- | --- | --- | --- | --- | --- | --- | --- | --- | --- | --- | --- | --- | --- | --- | --- | --- | --- | --- | --- |
| **Sample** | **Follow- up sample (sex)** | **Outcome** | **Follow-up (years)** | | **Baseline age (years)** | | **CV risk score** | | **Cognitive assessments or dementia criteria** | | **Outcome measure** | | **Results** | | **Multivariate adjustment** | **General conclusion** | |  | | |
| Framingham models | | |  | |  | |  | |  | |  | |  | |  |  | |  | | |
| Normative Aging Study | 235 (all men) | Change in cognition scores | 3 | | Mean 66.4 (sd: 6.7) | | Modified FSRP (omitted age) | | Category Fluency, DSB, WLIR, WLDR, PC | | β coefficient | | β (standard error); standardised β; change in R² Category Fluency -0.24 (0.11); -0.12***; 0.01*, DSB -0.09 (0.05); -0.10; 0.00, WLIR -0.01 (0.03); -0.02; 0.00, WLDR 0.00 (0.04); 0.00; 0.00, PC -0.02 (0.03); -0.03; 0.00 | | Age, education and baseline cognitive performance | Greater stroke risk is associated with greater decline in verbal fluency performance and stroke risk exhibits a greater relation with fluency decline than with decline in other cognitive abilities such as memory. | |  | | |
| English Longitudinal Study of Ageing | 8780 (men 3951, women 4829) | Change in cognition scores | 4 | | Mean 62.5 | | Modified FSRP (LVH excluded) and Framingham CVD scores. | | Cognitive Index, Memory Index, Executive Index | | β coefficient | | FSRP and Framingham CVD results detailed below | | Gender, age, alcohol, physical activity, educational qualifications, depression and baseline cognitive function | FSRP and Framingham CVD Score were significantly associated with cognitive decline on both global and specific measures at 4 year follow-up. | |  | | |
|  |  |  |  | |  | |  | |  | |  | | FSRP β (95% CI) | |  |  | |  | | |
|  |  |  |  | |  | |  | |  | |  | | Cognitive Index: | |  |  | |  | | |
|  |  |  |  | |  | |  | |  | |  | | Lower= ref | |  |  | |  | | |
|  |  |  |  | |  | |  | |  | |  | | Second= 0.06 (-0.34, 0.47) | |  |  | |  | | |
|  |  |  |  | |  | |  | |  | |  | | Third= -0.61 (-1.10, -0.12)* | |  |  | |  | | |
|  |  |  |  | |  | |  | |  | |  | | Upper= -0.73 (-1.37, -0.10)* | |  |  | |  | | |
|  |  |  |  | |  | |  | |  | |  | | Memory Index: | |  |  | |  | | |
|  |  |  |  | |  | |  | |  | |  | | Lower= ref | |  |  | |  | | |
|  |  |  |  | |  | |  | |  | |  | | Second= -0.02 (-0.30, 0.27) | |  |  | |  | | |
|  |  |  |  | |  | |  | |  | |  | | Third= -0.50 (-0.84, -0.16)* | |  |  | |  | | |
|  |  |  |  | |  | |  | |  | |  | | Upper= -0.56 (-0.99, -0.12)* | |  |  | |  | | |
|  |  |  |  | |  | |  | |  | |  | | Executive Index: | |  |  | |  | | |
|  |  |  |  | |  | |  | |  | |  | | Lower= ref | |  |  | |  | | |
|  |  |  |  | |  | |  | |  | |  | | Second= -0.03 (-0.27, -0.22) | |  |  | |  | | |
|  |  |  |  | |  | |  | |  | |  | | Third= -0.23 (-0.52, 0.06) | |  |  | |  | | |
|  |  |  |  | |  | |  | |  | |  | | Upper= -0.37 (-0.74, -0.01)* | |  |  | |  | | |
|  |  |  |  | |  | |  | |  | |  | | Framingham CVD Score β (95% CI) | |  |  | |  | | |
|  |  |  |  | |  | |  | |  | |  | | Cognitive Index: | |  |  | |  | | |
|  |  |  |  | |  | |  | |  | |  | | Lower= ref | |  |  | |  | | |
|  |  |  |  | |  | |  | |  | |  | | Second= 0.41 (-0.84, 0.02) | |  |  | |  | | |
|  |  |  |  | |  | |  | |  | |  | | Third= -0.72 (-1.234, -0.20)* | |  |  | |  | | |
|  |  |  |  | |  | |  | |  | |  | | Upper= -0.92 (-1.53, -0.31)* | |  |  | |  | | |
|  |  |  |  | |  | |  | |  | |  | | Memory Index: | |  |  | |  | | |
|  |  |  |  | |  | |  | |  | |  | | Lower= ref | |  |  | |  | | |
|  |  |  |  | |  | |  | |  | |  | | Second= -0.23 (-0.52, 0.07) | |  |  | |  | | |
|  |  |  |  | |  | |  | |  | |  | | Third= -0.59 (-0.94, -0.24)* | |  |  | |  | | |
|  |  |  |  | |  | |  | |  | |  | | Upper= -0.56 (-0.99, -0.13)* | |  |  | |  | | |
|  |  |  |  | |  | |  | |  | |  | | Executive Index: | |  |  | |  | | |
|  |  |  |  | |  | |  | |  | |  | | Lower= ref | |  |  | |  | | |
|  |  |  |  | |  | |  | |  | |  | | Second= -0.21 (-0.47, -0.06) | |  |  | |  | | |
|  |  |  |  | |  | |  | |  | |  | | Third= -0.23 (-0.54, 0.07) | |  |  | |  | | |
|  |  |  |  | |  | |  | |  | |  | | Upper= -0.45 (-0.80, -0.08)* | |  |  | |  | | |
| Whitehall II Study | 4827 (men 3486, women 1341) | Change in cognition scores | 10 | | Mean (sd: 55 (6) | | Framingham CVD score (10% point increment associations) | | Reasoning (AH4-I), Memory, Semantic Fluency, Phonemic Fluency, Vocabulary (Mill Hill) | | β coefficient | | Men β (95% CI) MANOVA*: Reasoning (AH4-I) -0.47 (-0.82, -0.11)**, Memory 0.06 (-0.09, 0.21) (ns), Semantic fluency -0.15 (-0.35, 0.04) (ns), Phonemic fluency -0.16 (-0.38, 0.05) (ns), Vocabulary (Mill Hill) -0.08 (-0.21, 0.04) (ns) Women β (95% CI) MANOVA*: Reasoning (AH 4-I) 1.17 (-0.08, 2.44) (ns), Memory -0.27 (-0.91, 0.36) (ns) , Semantic fluency -0.67 (-1.36, 0.02) (ns), Phonemic fluency -0.08 (-0.89, 0.72) (ns) , Vocabulary (Mill Hill) -0.42 (-0.89, 0.04) (ns) | | Age, ethnicity, marital status, education | In multivariate adjusted models, 10% higher CVD risk was associated with greater overall 10 year cognitive decline in men, reasoning in particular. | |  | | |
| Whitehall II Study | CVD and CAIDE risk score n=4374 (men 3162, women 1212) FSRP and CAIDE risk score n=5157 (men 3651, women 1506) | Change in cognition scores | 10 | | Mean 55.6 | | Framingham CVD score and FSRP (CAIDE shown in separate table) (low, intermediate and high risk groups (<7, 7 to <13, and >=13), stroke (<4, 4 to <6, and >=6), and dementia (<7, 7 to 8, and >=9)) | | Reasoning (AH4-I), 20-word free Recall test (verbal memory), Phonemic verbal fluency, Semantic verbal fluency, Mill Hill Vocabulary test,Global cognition (from all 5 tests) | | β coefficient | | Framingham CVD Score β (95% CI) Reasoning: Low -0.26 (-0.29, -0.23); Intermediate -0.31 (-0.34, -0.28); High -0.36 (-0.41, -0.38)*** Standardized risk -0.06 (-0.08, -0.04)***; Memory: Low -0.20 (-0.25, -0.15); Intermediate -0.29 (-0.34, -0.24); High -0.27 (-0.32, -0.21) Standardized risk -0.03 (-0.06, 0.00); Phonemic fluency: Low -0.31 (-0.35, -0.27); Intermediate -0.36 (-0.40, -0.32); High -0.39 (-0.44, -0.35)** Standardized risk -0.03 (-0.06, -0.01)**; Semantic fluency: Low -0.31 (-0.35, -0.27); Intermediate -0.36 (-0.40, -0.32); High -0.39 (-0.44, -0.35)*** Standardized risk -0.05 (-0.07, -0.02)***; Vocabulary: Low 0.05 (0.03, 0.08); Intermediate 0.03 (0.002, 0.05); High -0.02 (-0.05, 0.001)*** Standardized risk -0.04 (-0.05, -0.03)***; Global: Low -0.26 (-0.28, -0.23); Intermediate -0.34 (-0.37, -0.32); High -0.40 (-0.43, -0.37)*** Standardized risk -0.06 (-0.08, -0.05)*** | | None | The CAIDE and Framingham risk scores predict cognitive decline in late middle age, but the Framingham risk scores may have an advantage over the CAIDE risk score for assessing risk of cognitive decline. | |  | | |
|  |  |  |  | |  | |  | |  | |  | | FSRP β (95% CI) | |  |  | |  | | |
|  |  |  |  | |  | |  | |  | |  | | Reasoning: Low -0.27 (-0.29, -0.24); Intermediate -0.34 (-0.36, -0.31); High 0.42 (-0.45, -0.38)*** Standardized risk -0.05 (-0.06, -0.03)***; Memory: Low -0.24 (-0.28, -0.20); Intermediate -0.27 (-0.31, -0.22); High -0.25 (-0.32, -0.19) Standardized risk -0.03 (-0.06, 0.00); Phonemic fluency: Low -0.32 (-0.36, -0.29); Intermediate -0.36 (-0.39, -0.32); High -0.42 (-0.47, -0.37)** Standardized risk -0.03 (-0.06, -0.01)**; Semantic fluency: Low -0.26 (-0.29, -0.22); Intermediate -0.33 (-0.37, -0.29); High-0.40 (-0.44, -0.34)*** Standardized risk 0.05 (-0.08, -0.03)***; Vocabulary: Low 0.04 (0.03, 0.07); Intermediate 0.02 (-0.001, 0.04); High -0.05 (-0.08, -0.02)*** Standardized risk -0.04 (-0.05, -0.02)***; Global: Low 0.21 (-0.23, -0.19); Intermediate -0.26 (-0.28, -0.24); High 0.31 (-0.34, -0.29)*** Standardized risk -0.04 (-0.05, -0.03)*** | |  |  | |  | | |
| Whitehall II Study | 5810 (men 4153, women 1657) | Changes in cognition scores | 10 | | Mean 55.6 | | FSRP (4 quartiles) | | Reasoning (AH4-I), 20-word free recall test (verbal memory), Phonemic verbal fluency, Semantic verbal fluency, Mill Hill Vocabulary test, Global cognition (from all 5 tests) | | β coefficient | | β (95% CI) 4 quartiles Reasoning: 1st (ref)-0.33 (-0.36, -0.29); 2nd -0.32 (-0.36, -0.28); 3rd -0.35 (-0.40, -0.30); 4th -0.35 (-0.40, 0.30); Memory: 1st (ref) -0.25 (-0.32, -0.29); 2nd -0.20 (-0.28, -0.12); 3rd -0.25 (-0.34, -0.16); 4th -0.18 (-0.27, -0.009); Phonemic fluency: 1st (ref) -0.33 (-0.39, 0.27); 2nd 0.34 (-0.41, 0.28); 3rd -0.39 (-0.47, -0.32); 4th -0.42 (-0.48, -0.13)*; Semantic fluency: 1st (ref) -0.21 (-0.27, -0.16); 2nd -0.27 (-0.33, -0.20); 3rd -0.25 (-0.32,0.18); 4th -0.29 (-0.36, -0.22)*; Vocabulary: 1st (ref) 0.04 (0.01, 0.08); 2nd 0.05 (0.01, 0.09); 3rd 0.03 (0.01, 0.08); 4th (-0.01 (-0.05, 0.03)**; Global cognition: 1st (ref) -0.21 (-0.24, -0.19); 2nd -0.16 (-0.24, -0.18); 3rd -0.24 (-0.27,-0.20); 4th (-0.25 (-0.28, -0.21)* | | Age, sex, ethnicity and education, depressive symptoms, physical activity and alcohol use | Higher risk of stroke was associated with faster decline in phonemic and semantic fluency, vocabulary and global cognition in the highest risk quartile compared to the lowest risk quartile. | |  | | |
| Reasons for Geographic and Racial Differences in Stroke (REGARDS) | 23830 | Cognitive impairment incidence | 4 | | Mean 64.2 | | FSRP | | Global cognition (SIS <5 indicates cognitive impairment) | | OR | | Risk of incident cognitive impairment OR (95% CI) High stroke risk (relative to lower risk FSRP >=20) Blacks: 2.12 (1.77, 2.53) Whites: 3.10 (2.60, 3.69) | | Sex, education, race, income and SES | After adjustment for demographics and SES, a higher FSRP was associated with increased risk of incident cognitive impairment. | |  | | |
| Rancho Bernardo Study | 985 (men 394, women 591) | Changes in cognition scores | Median 9.8 (IQR: 2.5, 17.4) | | Mean 66.8 (sd: 8.5) | | Framingham CHD score (risk categories low <10%; intermediate (10-20%) and high (>20%); Women also stratified by tertiles as small number high risk | | MMSE, Trails B, Verbal Fluency, Short term recall, Long term recall | | β coefficient | | Men risk score categories β, linear low =ref MMSE: intermediate 0.017; high 0.017; Trails B: intermediate 0.237; high 0.804; Verbal fluency: intermediate 0.026; high 0.026; Short term recall: intermediate 0.090; high 0.047; Long recall: intermediate 0.047; High 0.045 Women risk score categories β, linear and quad low =ref. MMSE: intermediate 0.086, quad -0.008* high 0.276**, quad -0.020**; Trails B: intermediate -3.200*, quad 0.402**; high -2.685, quad 0.327; Verbal fluency: intermediate -0.080, quad 0.004; high -0.637**, quad 0.047**; Short term recall: intermediate 0.020; high 0.102; Long term recall: intermediate -0.301, quad 0.009; High -1.442*, quad 0.124** | | Baseline age, education and number of cognitive assessments | For older women, very low levels of CHD risk were associated with prevention of cognitive function over time. | |  | | |
|  |  |  |  | |  | |  | |  | |  | | Women tertiles β, linear and quad 1=ref. MMSE: 2= -0.015, quad -0.001; 3= 0.120*, quad -0.011**; Trails B: 2= -1.634, quad 0.196*; 3= 3.542**, quad 0.444**; Verbal fluency: 2= -0.167, quad 0.010; 3= -0.270*, quad 0.018*; Short term recall: 2=0.048; 3= 0.055; Long term recall: 2=-1.069**, quad 0.067*; 3= -1.010**, quad 0.065* | |  |  | |  | | |
| Reasons for Geographic and Racial Differences in Stroke (REGARDS) study | 23752 | Incident cognitive impairment | Mean 4.1 | | Mean 64.3 | | FSRP (results based on each sd higher baseline FSRP score) | | Decline from baseline cognitive score of 5 or 6 (of possible 6 points) to the most recent follow-up score of 4 or less on the SIS | | OR | | OR (95% CI): 1.41 (1.37, 1.46) | | Sex, race, region and education | Total FSRP score predicts development of clinically significant cognitive dysfunction. | |  | | |
| CAIDE models | |  |  | |  | |  | |  | |  | |  | |  |  | |  | | |
| Hoorn Study | 322 | Cognitive impairment | 15 | | 50-64. Mean (sd) 55.9 (3.7) | | CAIDE Model 1 | | Information-processing speed (Trail Making test A, Stroop Color-Word test parts, Digit Symbol test of the WAIS-III), Attention and executive functioning (Trail Making test B, Stroop Color-Word test, Brixton Spatial Anticipation test, Verbal Fluency), Visuoconstruction (copy trial of the Rey-Osterrieth Complex Figure), abstract reasoning (Raven Progressive Matrices), language (short form of the Token test) and memory (WAIS-III, Corsi Block-Tapping task, Rey Auditory Verbal Learning test, Location Learning test, and the Rey Osterrieth Complex Figure) | | OR | | OR (95% CI) per point increase in RF sum; Information-processing speed 1.22 (1.01, 1.46)*; Attention and executive functioning 1.17 (0.99, 1.38); Visuoconstruction 1.32 (1.02, 1.71)*; Abstract reasoning 1.40 (1.06, 1.84)*; Language 1.08 (0.82, 1.42); Memory 0.87 (0.74, 1.02) OR (95% CI) with RF sum score >=9: Information-processing speed 3.07 (1.37, 6.90)*; Attention and executive functioning 1.30 (0.66, 2.56); Visuoconstruction 1.74 (0.61, 5.02); Abstract reasoning 3.97 (1.07, 14.71)*; Language 1.33 (0.44, 4.07); Memory 0.66 (0.34, 1.29) OR (95% CI) per point increase in sum score of modifiable RF: Information-processing speed 1.22 (0.99, 1.51); Attention and executive functioning 126 (1.04, 1.54)*; Visuoconstruction 1.26 (0.94, 1.69); Abstract reasoning 1.25 (0.91, 1.71); Language 1.09 (0.79, 1.51); Memory 0.84 (0.68, 1.03) | | Age, sex and education | The CAIDE score predicts late life cognitive impairment (for information processing speed, visuoconstruction and abstract reasoning). | |  | | |
| Whitehall II Study | CVD and CAIDE risk score n=4374 (men 3162, women 1212) FSRP and CAIDE risk score n=5157 (men 3651, women 1506) | Change in cognition scores | 10 | | Mean 55.6 | | Framingham CVD score, FSRP, CAIDE Model 1 and CAIDE Model 2 (low, intermediate and high risk groups (<7, 7 to <13, and >=13), stroke (<4, 4 to <6, and >=6), and dementia (<7, 7 to 8, and >=9) | | Reasoning (AH4-I), 20-word free recall test (verbal memory), Phonemic verbal fluency, Semantic verbal fluency, Mill Hill Vocabulary test, Global cognition (from all 5 tests) | | β coefficient | | CAIDE and Framingham CVD Score results CAIDE Model 1 β (95% CI): Reasoning: Low -0.28 (-0.31, -0.26) Intermediate -0.35 (-0.38, -0.32) High -0.36 (-0.39, -0.33) P trend*** Standardized risk -0.05 (-0.06, -0.03)***; Memory: Low -0.24 (-0.28, -0.19) Intermediate -0.27 (-0.33, -0.22) High -0.26 (-0.32, -0.19) P trend ns Standardized risk -0.01 (-0.04, 0.01)ns; Phonemic fluency: Low -0.34 (-0.38, -0.31) Intermediate -0.37 (-0.42, -0.33) High -0.36 (-0.41, -0.31) P trend ns Standardized risk -0.01 (-0.04, 0.01)ns; Semantic fluency: Low -0.29 (-0.33, -0.26) Intermediate -0.32 (-0.37, -0.28) High -0.29 (-0.35, -0.24) P trend ns Standardized risk 0.001 (-0.02, 0.02)ns; Vocabulary: Low 0.05 (0.03, 0.07) Intermediate 0.004 (-0.02, 0.03) High -0.02 (-0.05, 0.01) P trend*** Standardized risk -0.02 (-0.04, -0.01)**; Global: Low -0.31 (-0.33, -0.28) Intermediate -0.36 (-0.39, -0.34) High -0.35 (-0.39, -0.32) P trend ** Standardized risk -0.03 (-0.04, -0.01)** | | None | The CAIDE and Framingham risk scores predict cognitive decline in late middle age, but the Framingham risk scores may have an advantage over the CAIDE risk score for assessing risk of cognitive decline. | |  | | |
|  |  |  |  | |  | |  | |  | |  | | CAIDE Model 2 β (95% CI): Reasoning: Low -0.27 (-0.30, -0.25) Intermediate -0.35 (-0.38, -0.32) High -0.43 (-0.47, -0.39) P trend*** Standardized risk -0.06 (-0.08,-0.05)***; Memory: Low -0.23 (-0.28, -0.18) Intermediate -0.30 (-0.36, -0.25) High -0.28 (-0.36, -0.21) P trend ns Standardized risk -0.03 (-0.06, 0.00)ns; Phonemic fluency: Low -0.35 (-0.39, -0.32) Intermediate -0.37 (-0.41, -0.32) High -0.39 (-0.44, -0.33) P trend ns Standardized risk -0.02 (-0.04, 0.01)ns; Semantic fluency: Low -0.30 (-0.35, -0.28) Intermediate -0.31 (-0.36, -0.27) High -0.31 (-0.35, -0.28) P trend ns Standardized risk 0.01 (-0.02, 0.03)ns; Vocabulary: Low 0.06 (0.04, 0.08) Intermediate 0.003 (-0.02, 0.03) High -0.04 (-0.07, -0.004) P trend*** Standardized risk -0.03 (-0.04, 0.01)**; Global: Low -0.30 (-0.32, -0.27) Intermediate -0.36 (-0.39, -0.34) High -0.39 (-0.43, -0.35) P trend *** Standardized risk -0.04 (-0.05, -0.02)** | |  |  | |  | | |
|  |  |  |  | |  | |  | |  | |  | | CAIDE and FSRP CAIDE Model 1 β (95% CI) Reasoning: Low -0.28 (-0.30, -0.26) Intermediate -0.35 (-0.38, -0.32) High -0.37 (-0.40, -0.33) P trend*** Standardized risk -0.05 (-0.06, -0.04)***; Memory: Low -0.24 (-0.28, -0.20), Intermediate -0.27 (-0.32, -0.22), High -0.27 (-0.33, -0.20) P trend ns Standardized risk -0.02 (-0.04, 0.01)ns; Phonemic fluency: Low -0.34 (-0.37, -0.30) Intermediate -0.37 (-0.41, -0.33) High -0.37 (-0.41, -0.31) P trend ns Standardized risk -0.02 (-0.04, 0.01)ns; Semantic fluency: Low -0.29 (-0.32, -0.26) Intermediate -0.34 (-0.38, -0.30) High -0.30 (-0.35, -0.26) P trend ns Standardized risk -0.01 (-0.03, 0.01)ns; Vocabulary: Low 0.05 (0.03, 0.07) Intermediate 0.006 (-0.02, 0.03) High -0.03 (-0.06, -0.002) P trend***Standardized risk -0.03 (-0.04, -0.01)***; Global: Low -0.22 (-0.24, -0.21) Intermediate -0.27 (-0.29, -0.25) High -0.27 (-0.29, -0.24) P trend*** Standardized risk -0.02 (-0.03, -0.01)*** | |  |  | |  | | |
|  |  |  |  | |  | |  | |  | |  | | CAIDE Model 2 β (95% CI): Reasoning: Low -0.26 (-0.29, -0.24) Intermediate -0.35 (-0.38, -0.32) High -0.42 (-0.46, -0.39) P trend***  Standardized risk -0.07 (-0.08, -0.05)***; Memory: Low -0.23 (-0.28, -0.19) Intermediate -0.28 (-0.33, -0.23) High -0.29 (-0.36, -0.22) P trend ns Standardized risk -0.02 (-0.05, 0.00)ns; Phonemic fluency: Low -0.34 (-0.38, -0.31) Intermediate -0.37 (-0.41, -0.33) High -0.39 (-0.45, -0.34) P trend ns Standardized risk -0.02 (-0.05, 0.00)ns; Semantic fluency: Low -0.31 (-0.34, -0.28); Intermediate -0.33 (-0.37, -0.29) High -0.33 (-0.38, -0.27) P trend ns Standardized risk -0.01 (-0.03, 0.02)ns; Vocabulary: Low 0.06 (0.04, 0.08) Intermediate 0.01 (-0.01, 0.03) High -0.05 (-0.08, -0.01) P trend*** Standardized risk -0.03 (-0.04, -0.01)***; Global: Low -0.22 (-0.24, -0.20) Intermediate -0.26 (-0.28, -0.25)  High -0.30 (-0.32, -0.27) P trend*** Standardized risk -0.03 (-0.04, -0.02)*** | |  |  | |  | | |
| The older Finnish Twin Cohort | 2165 (men 1107, women 1058) | Moderate to severe cognitive impairment | Mean: 22.6 (sd: 2.3) | | Mean: 51.7 (sd: 6.1) | | CAIDE Model 1 and CAIDE Model 2 | | TELE | | OR and AUC | | 328 participants developed either moderate or severe cognitive impairment over time CAIDE Model 1, OR (95% CI): Quartile I (0-5), 1.00 Quartile II (6-7), 2.23 (0.80, 6.18) Quartile III (8), 6.81 (2.56, 18.1)* Quartile IV (9-15), 10.4 (4.11, 26.1)* | | Follow-up | Strong association between increasing risk score and risk of cognitive impairment. | |  | | |
|  |  |  |  | |  | |  | |  | |  | | CAIDE Model 2, OR (95% CI): Quartile I (0-6), 1.00 Quartile II (7-8), 0.66 (0.15, 2.96) Quartile III (9-10), 4.66 (1.61, 13.5)* Quartile IV (11-18), 8.56 (3.06, 24.0)* | |  |  | |  | | |
|  |  |  |  | |  | |  | |  | |  | | CAIDE Model 1 AUC (95% CI) 0.74 (0.69, 0.79) | |  |  | |  | | |
|  |  |  |  | |  | |  | |  | |  | | CAIDE Model 2 AUC (95% CI) 0.75 (0.70, 0.81) | |  |  | |  | | |
| Other cardiovascular risk models | | |  | |  | |  | |  | |  | |  | |  |  | |  | | |
| Baltimore Study of Black Aging | 435 (men 106, women 329) | Change in cognitive test scores | 2.5 | | Mean 66.8 (sd: 9.0) | | Vascular risk factor index | | MMSE, DSST | | β coefficient | | MMSE 0.02 ns; Digit Symbol -0.07* | | None | Higher vascular risk associated with a decrease in processing speed, but no effect seen for global cognition | |  | | |
| Cardiovascular Health Study | 3832 | Changes in cognitive test scores | 5 | | 65+ | | CHS Stroke risk score (Quartiles) | | At least 1 point/year on 3MSE and 2 points/year on DSST | | OR | | 3MSE OR (95% CI) (No incident stroke or TIA) 1=ref (1.00) 2=1.03 (0.77, 1.39) 3=1.43 (1.05, 1.94) 4=1.60 (1.16, 2.21) linear test of trend p value***. 3MSE OR (95% CI) (No MRI infarct at baseline or follow-up and no change in white matter grade) 1=ref (1.00) 2=1.24 (0.57, 2.69) 3=1.38 (0.57, 3.34) 4=1.70 (0.65, 4.43) linear test of trend p value ns | | Baseline cognitive test score, age, age squared, gender, race, education, income and ApoE status | Stroke risk score was a predictor of cognitive decline in the general cohort. | |  | | |
|  |  |  |  | |  | |  | |  | |  | | DSST OR (95% CI) (No incident stroke of TIA) 1=ref (1.00) 2=1.11 (0.82, 1.49) 3=1.63 (1.20, 2.21) 4=1.95 (1.41, 2.70) linear test of trend p value*** DSST OR (95% CI) (No MRI infarct at baseline or follow-up and no change in white matter grade) 1=ref (1.00) 2=0.83 (0.36, 1.91) 3=1.82 (0,75, 4.42) 4=3.02 (1.15, 7.94) linear test of trend p value* | |  |  | |  | | |
|  |  |  |  | |  | |  | |  | |  | | Also split by high cognitive function at baseline (present/absent): 3MSE OR (95% CI) (Present) 1=ref (1.00) 2=0.81 (0.51, 1.28) 3=1.38 (0.88, 2.18) 4=0.95 (0.57, 1.58) linear test of trend p value ns (Absent): 1=ref (1.00) 2=1.27 (0.86, 1.88) 3=1.60 (1.08, 2.37) 4=2.11 (1.42, 3.13) linear test of trend p value*** | |  |  | |  | | |
|  |  |  |  | |  | |  | |  | |  | | DSST OR (95% CI) (Present): 1=ref (1.00) 2=0.75 (0.47, 1.19) 3=1.55 (0.99, 2.42) 4=1.76 (1.09, 2.85) linear test of trend p value*** (Absent): 1=ref (1.00) 2=1.33 (0.91, 1.94) 3=1.59 (1.08, 2.34) 4=1.83 (1.23, 2.72) linear test of trend p value*** | |  |  | |  | | |
| Coronary Artery Risk Development in Young Adults (CARDIA) Study | 2932 (men 1308, women 1624) | Changes in cognitive test scores | 25 | | 18-30 | | Ideal cardiovascular metrics | | DSST, Stroop test, RAVLT | | β coefficient | | DSST Mean (95% CI) per each additional ideal component Year 25: 0.94 (0.55, 1.34) p for trend*** Mean (95% CI) ≥5 components present at: No exams: 67.8 (67.0, 68.7) 1 exam: 69.0 (68.0, 69.9) 2 exams: 70.1 (69.0, 71.3) All 3 exams: 71.4 (69.8, 73.1) p for trend*** | | Age, sex, race (black, white), educational attainment, alcohol use and study centre | Ideal cardiovascular health was associated with better psychomotor speed, executive function and verbal memory in midlife. | |  | | |
|  |  |  |  | |  | |  | |  | |  | | Stroop test Mean (95% CI) per each additional ideal component Year 25: -0.40 (-0.69, -0.11) p for trend*** Mean (95% CI) ≥5 components present at: No exams: 23.8 (23.2, 24.4) 1 exam: 23.0 (22.3, 23.7) 2 exams: 22.5 (21.7, 23.3) All 3 exams: 21.8 (69.8, 73.1) p for trend*** | |  |  | |  | | |
|  |  |  |  | |  | |  | |  | |  | | RAVLT Mean (95% CI) per each additional ideal component Year 25: 0.12 (0.04, 0.20) p for trend** Mean (95% CI) ≥5 components present at: No exams: 8.0 (7.8, 8.1) 1 exam: 8.3 (8.1, 8.5) 2 exams: 8.2 (8.0, 8.5) All 3 exams: 8.5 (8.2, 8.8) p for trend** | |  |  | |  | | |
|  |  |  | |  | |  | |  |  |  | |  | |  | |  |  | |  | |

Abbreviations: 3MSE, Modified Mini-Mental State Exam; AD, Alzheimer’s disease; AH4-I, Alice Heim 4-I; ApoE, Apolipoprotein E; CAIDE, Cardiovascular Risk Factors, Aging and Dementia; CI, confidence interval; CIND, cognitive impairment no dementia; CHD, coronary heart disease; CVD, cardiovascular disease; DSB, Digit Span Backward; DSM-IV, Diagnostic and Statistical Manual for Mental Disorders-IV; FSRP, Framingham Stroke Risk Profile; HR, hazard ratio; IQR, inter-quartile range; LVH, left-ventricular hypertrophy; MANOVA, Multivariate Analysis of Variance; MCI, mild cognitive impairment; MMSE, Mini-Mental State Examination; NINCDS-ADRDA, National Institute of Neurological and Communicative Disorders and Stroke-Alzheimer Disease and Related Disorders Association; OR, odds ratio; PC, pattern comparison; ref, reference category; sd, standard deviation; SEVLT, Spanish and English Verbal Learning Test; SES, socio-economic status; SIS, Six-item Screener; Trails B, Trail Making Test B; WLDR, word list delayed recall; WLIR, word list immediate recall

* p<0.05 **p<0.01 ***p<0.001 ns=non-significant

**References**

1. Brady CB, Spiro A, McGlinchey-Berroth R, Milberg W, Gaziano JM (2001) Stroke risk predicts verbal fluency decline in healthy older men: Evidence from the normative aging study. Journals of Gerontology Series B-Psychological Sciences and Social Sciences 56: P340-P346.

2. Dregan A, Stewart R, Gulliford MC (2013) Cardiovascular risk factors and cognitive decline in adults aged 50 and over: a population-based cohort study. Age Ageing 42: 338-345.

3. Kaffashian S, Dugravot A, Nabi H, Batty GD, Brunner E, et al. (2011) Predictive utility of the Framingham general cardiovascular disease risk profile for cognitive function: evidence from the Whitehall II study. Eur Heart J 32: 2326-2332.

4. Kaffashian S, Dugravot A, Elbaz A, Shipley MJ, Sabia S, et al. (2013) Predicting cognitive decline: a dementia risk score vs. the Framingham vascular risk scores. Neurology 80: 1300-1306.

5. Kaffashian S, Dugravot A, Brunner EJ, Sabia S, Ankri J, et al. (2013) Midlife stroke risk and cognitive decline: a 10-year follow-up of the Whitehall II cohort study. Alzheimers & Dementia 9: 572-579.

6. Kelley BJ, McClure LA, Letter AJ, Wadley VG, Unverzagt FW, et al. (2013) Report of stroke-like symptoms predicts incident cognitive impairment in a stroke-free cohort. Neurology 81: 113-118.

7. Laughlin GA, McEvoy LK, von Mullen D, Daniels LB, Kritz-Silverstein D, et al. (2011) Sex Differences in the Association of Framingham Cardiac Risk Score With Cognitive Decline in Community-Dwelling Elders Without Clinical Heart Disease. Psychosomatic Medicine 73: 683-689.

8. Unverzagt FW, McClure LA, Wadley VG, Jenny NS, Go RC, et al. (2011) Vascular risk factors and cognitive impairment in a stroke-free cohort. Neurology 77: 1729-1736.

9. Reijmer YD, van den Berg E, van Sonsbeek S, Dekker JM, Nijpels G, et al. (2011) Dementia risk score predicts cognitive impairment after a period of 15 years in a nondemented population. Dement Geriatr Cogn Disord 31: 152-157.

10. Virta JJ, Heikkila K, Perola M, Koskenvuo M, Raiha I, et al. (2013) Midlife cardiovascular risk factors and late cognitive impairment. Eur J Epidemiol 28: 405-416.

11. Carmasin JS, Mast BT, Allaire JC, Whitfield KE (2014) Vascular risk factors, depression, and cognitive change among African American older adults. Int J Geriatr Psychiatry 29: 291-298.

12. Elkins JS, O'Meara ES, Longstreth WT, Jr., Carlson MC, Manolio TA, et al. (2004) Stroke risk factors and loss of high cognitive function. Neurology 63: 793-799.

13. Reis JP, Loria CM, Launer LJ, Sidney S, Liu K, et al. (2013) Cardiovascular health through young adulthood and cognitive functioning in midlife. Annals of Neurology 73: 170-179.
