# Supplementary material for: Cardiovascular Disease Risk Models and Longitudinal Changes in Cognition: A Systematic Review
Source: PLoS One. 2014 Dec 5;9(12):e114431. doi: 10.1371/journal.pone.0114431 (PMC4257686; doi:10.1371/journal.pone.0114431)
Supplement: Materials S1 — Sample search strategy. (DOCX) [file pone.0114431.s004.docx]

**Sample search strategy**

1. (cognit* OR dementia OR Alzheimer*). ab, ti.
2. (Framingham OR qrisk* OR caide). ab, ti.
3. 1 AND 2
4. (vascular risk). ab, ti.
5. (stroke risk).ab, ti.
6. (cardiovascular risk). ab, ti.
7. (cardiovascular health). ab, ti.
8. 4 OR 5 OR 6 OR 7
9. 1 AND 8
10. 3 OR 9
11. Limit 10 to English language and humans
